# Binge eating disorder and night eating syndrome in adults with type 2 diabetes: a systematic review

**DOI:** 10.1186/s40337-018-0223-1

**Published:** 2018-11-06

**Authors:** Sally Abbott, Naomi Dindol, Abd A. Tahrani, Milan K. Piya

**Affiliations:** 1 Department of Diabetes and Endocrinology, University Hospitals Birmingham NHS Foundation Trust, Birmingham, UK; 2 Institute of Metabolism and Systems Research, University of Birmingham, Birmingham, UK; 3 Centre of Endocrinology and Diabetes and Metabolism, Birmingham Health Partners, Birmingham, UK; 4 Macarthur Clinical School, School of Medicine, Western Sydney University, Campbelltown, NSW Australia; 5 Macarthur Diabetes Service, Camden and Campbelltown Hospitals, South Western Sydney Local Health District, Campbelltown, NSW Australia

## Abstract

**Background:**

Type 2 diabetes (T2DM) is increasing in prevalence worldwide, and is closely linked to obesity. Binge Eating Disorder (BED) and Night Eating Syndrome (NES) are eating disorders that are common in obesity, and may affect the management as well as long term outcomes of T2DM. Therefore, the aim of this review was to assess the prevalence and associations of BED or NES in adults with T2DM.

**Methods:**

We conducted a systematic review. The databases MEDLINE, CINAHL and AMED were searched for articles which met the inclusion criteria; including patients > 18 years old, with T2DM, and BED and/or NES. The reference lists of included studies were also searched. Meta-analysis was not attempted due to the limited number of studies that measured the outcomes of interest.

**Results:**

A total of 10 studies (2 included NES) were included in this systematic review. The number screened for BED and NES were 6527 and 1039 participants, respectively. Point prevalence was 1.2–8.0% for BED and 3.8–8.4% for NES. Patients with T2DM and BED had higher BMI than patients with T2DM without BED in the two studies that reported BMI. There was no statistically significant difference in HbA1c between patients with and without BED in the two studies that measured HbA1c.

**Conclusions:**

BED and NES are common in adults with T2DM, and BED is associated with higher BMI in patients with T2DM. However, only two studies reported important outcomes measures such as BMI and HbA1c in patients with T2DM. Hence, further well-designed studies are needed to assess the impact of BED and NES in patients with T2DM. Health Care Professionals should consider the diagnosis of BED and NES in patients with T2DM.

## Introduction

There is an increasing prevalence of diabetes worldwide, with over 400 million people living with Type 2 Diabetes Mellitus (T2DM), which is expected to rise to over 600 million by 2045 [1].The link between obesity and T2DM is well documented [2–6]. Obesity prevention and management is required to reduce the prevalence of T2DM, but the main aim of managing existing T2DM is to improve glycaemic control and cardiovascular disease risk factors to prevent long term micro and macro vascular complications [6, 7].

Binge Eating Disorder (BED) and Night Eating Syndrome (NES) are two eating disorders that are more common in people with obesity [8–10]. Bulimia Nervosa is another eating disorder that may occur in obesity and T2DM. Although not discussed in this article, Bulimia Nervosa has been covered in a previous review paper which found a prevalence of 0.3% among individuals with T2DM [11]. BED is characterised by consuming an objectively large amount of food over a discrete period of time; accompanied by a sense of loss of control and distress [12]. BED is prevalent in 1.9% to 2.8% of the general population [9], and people with BED are at 3–6 times greater risk of having obesity compared to those without BED [9]. A higher percentage of youths aged 10–17 years with clinical BED have also been found to have obesity compared to non-overeaters with a BMI above the 99th centile; 66.7% vs. 37.7%, (*p* < 0.05) [13]. NES describes nocturnal hyperphagia, insomnia and morning anorexia [14], and affects an estimated 1% of the general population [15], as high as 6–16% in patients with obesity [16, 17], and 2–20% of bariatric surgery patients [17].

Diagnosis of BED and NES is based on the DSM-5 criteria [12, 18], updated in 2013. The updated diagnostic criteria for BED and NES are described in Table 1. The most notable change was that BED was acknowledged as a separate diagnosis for the first time, and NES became listed as a specific disorder under Other Specified Feeding or Eating Disorder (OSFED). An individual can have a diagnosis of NES only if the behaviour is not better explained by another mental health disorder, such as BED. Therefore, BED and NES cannot coexist. Treatment rates for BED and NES are low and less than 40% of individuals with a lifetime diagnosis of BED have been treated [9]. This may be due to a lack of awareness of, or screening for BED and NES [19].

**Table 1:** Diagnostic Criteria

Eating Disorder	DSM-IV [18]	DSM-5 [12]
BED	*A* Recurrent episodes of binge eating. An episode of binge eating is characterised by both of the following: 1. eating, in a discrete period (e.g. within any 2-h period), an amount of food that is definitely larger than most people would eat in a similar period of time under similar circumstances 2. a sense of lack of control over eating during the episode	No change
	*B* The binge eating episodes are associated with > 3 of the following: 1. eating much more rapidly than normal 2. eating until feeling uncomfortably full 3. eating large amounts of food when not feeling physically hungry 4. eating alone because of being embarrassed by how much one is eating 5. feeling disgusted, depressed or very guilty after overeating	No change
	*C* Marked distress over binge eating is present	No change
	*D* The binge eating occurs on average 2 days per week for 6 months	The binge occurs, on average, more than once per week for 3 months
	*E* The binge eating is not associated with the regular use of inappropriate compensatory behaviours	The binge eating is not associated with **recurrent** use of inappropriate compensatory behaviour, such as in bulimia nervosa.
NES	NES would have been considered an eating disorder not otherwise categorised (EDNOS); but not listed as an eating disorder in its own right	NES is listed as an eating disorder under the other specified feeding or eating disorder (OSFED) category: 1. Recurrent episodes of night eating, as manifested by eating after awakening from sleep or by excessive food consumption after the evening meal 2. There is awareness and recall of eating 3. Night eating causes significant distress and/or impairment in functioning 4. The disordered pattern of eating is not better explained by binge eating disorder or another mental disorder, including substance use, and is not attributable to another medical disorder or to an effect of medication 5. The night eating is not better explained by external influences such as changes in the individual’s sleep–wake cycle or by local social norms

Previous studies have shown that patients with BED have higher prevalence of T2DM compared to general population matched controls, 15.2% vs. 2.2%, (OR 8.8, 95% CI 4.3–17.9) [20]. In addition, having BED increased the risk of incident T2DM compared to people without BED (RR 6.5, 95% CI 3.4–12.3) [20]. Similar results were found by Hudson et al. in which patients with BED were at increased risk of T2DM compared to those without BED (HR 1.7, 95% CI 1.1–2.6) despite adjustment for age, gender and BMI [21].

BED and NES might have a detrimental impact on the metabolic parameters in patients with T2DM. Increased severity of binge eating on a scale questionnaire has been shown to be significantly associated with increased risk of metabolic complications including raised HbA1C, blood pressure and BMI [22]. Experimental studies have shown that one-day of high-fat overfeeding in humans resulted in significant increases in postprandial glucose by 17.1%, and reductions in whole-body insulin sensitivity by 28% [23]. On the other hand, prescribed dietary components for T2DM management can also precipitate disordered eating, including dietary restraint and food preoccupation [24, 25]. In particular, very low calorie diets have been shown to significantly improve T2DM control and induce diabetes remission in 46% of patients with recent onset T2DM [26]. Therefore, more clinicians are advising low or very low calorie diets to patients who may have undiagnosed BED or NES. Furthermore, sleep deprivation and poor quality of sleep, which are characteristics of NES, are also risk factors for T2DM and obesity [27, 28].

Having BED or NES might have implications on the choice of treatment in patients with T2DM. For example, eating disorders might have an impact on the outcomes of bariatric surgery, while bariatric surgery might impact BED and NES [29–31].

Hence, we conducted a systematic review to assess the prevalence of BED and NES in patients with T2DM. We also assessed the relationships between BED/NES with BMI and HbA1c in patients with T2DM.

### Methodology

Inclusion criteria were studies of adults (> 18 years old), with known T2DM and reported data on prevalence of BED or NES. Diagnosis should have been made according to DSM-IV or DSM-5 criterion and using validated questionnaire or interview methods, outlined in Table 2. Studies based in eating disorder services were excluded, as these would have a large selection bias.

**Table 2:** Screening Methods

	Name	Outline
Questionnaire	Questionnaire on Eating and Weight Patterns (QEWP, QEWP-R)	A 27-item self-administered questionnaire that focuses on assessing diagnostic criteria for BED
	Eating Disorder Examination Questionnaire (EDE-Q)	A self-report version of the EDE interview. A 41-item self-reported questionnaire generating 4 subscales and a global score.
	Night Eating Questionnaire (NEQ)	A 14-item survey using 0–4 Likert responses to screen for symptoms of NES. Items are also used to distinguish NES from sleep-related eating disorder (SRED) and to confirm that symptoms have been present for > 3 months. Total NEQ scores may range from 0 to 52.
Interview	Eating Disorder Examination (EDE)	A semi-structured interview that provides a profile of psychopathology based 4 four subscales and a global score.
	Night Eating Syndrome History and Inventory (NESHI)	A semi-structured interview based on the NEQ that assesses the presence and frequency of night eating symptoms more comprehensively than the survey alone and allows for diagnosis of NES to be made.
	Structured Clinical Interview for DSM-IV Axis 1 (SCID-I)	A semi-structured interview to determine whether the individual meets the DSM-IV criteria for eating disorders.
	Structured Interview for Anorexic and Bulimic Syndromes, for Expert Rating (SIAB, SIAB-EX)	A semi-structured interview to assess for the specific and general psychopathology of eating disorders designed to establish diagnosis according to DSM-III-R.

Literature searches were made using keywords relevant to the framed question of this review. Literature searches were performed for studies from inception up to 26th January 2018. The databases MEDLINE, CINAHL and AMED were searched using the terms ‘Night Eating’ OR ‘Night Eating Syndrome’ OR ‘Binge Eating’ OR ‘Binge Eating Disorder’ OR ‘Eating Disorders’ AND ‘Diabetes’, ‘Diabetes Mellitus Type 2’, ‘Type 2 Diabetes’. To ensure all relevant literature was captured, the reference lists of the included primary studies were also searched.

The first two authors independently screened the titles and abstracts of identified studies. Studies meeting the eligibility criteria were then screened independently by both authors, using full-texts to determine inclusion. Any discrepancies were discussed in arbitration until a consensus was reached.

## Results

### Study selection

**Fig. 1:**
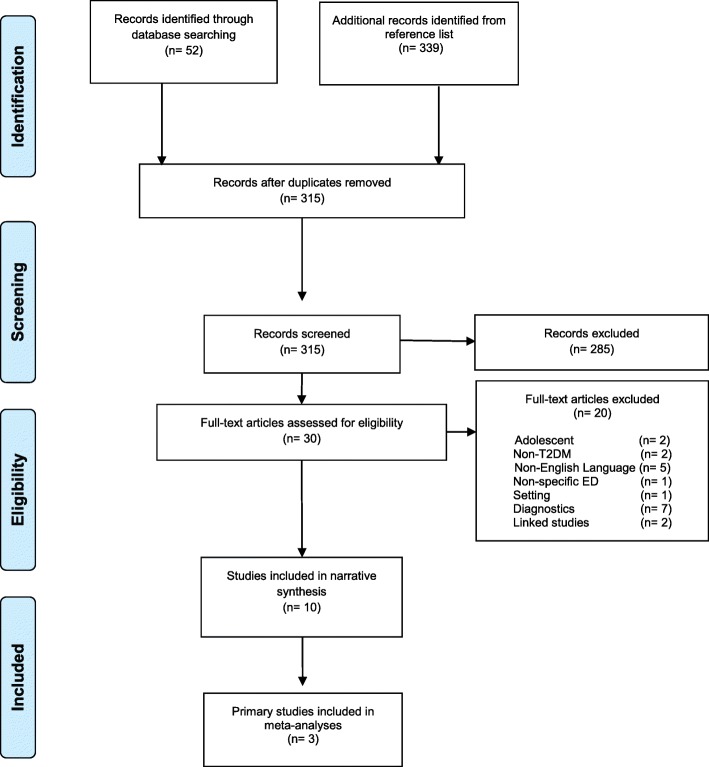
Flowchart of studies screened and included in the narrative synthesis and meta-analysis

The study selection process is detailed in Fig. 1. A total of 10 studies were identified for inclusion in the systematic review. These were generated from a search of the databases, which retrieved a total of 52 citations. Importing the reference lists of the 10 included studies provided an additional 339 citations. After adjusting for duplicates, the citations identified in the literature search totalled 315. Of those, 285 citations were discarded after screening their titles and abstracts as they did not meet the inclusion criteria. The full texts of the remaining 30 citations were then examined in further detail.

After examination of the full-text, 20 citations were excluded because they did not meet the inclusion criteria. The reasons for excluding studies are given in detail in Fig. 1.

### Study characteristics

The characteristics of included studies [32–41] are detailed in Table 3.

**Table 3:** Study Characteristics

Author	Country	Multi-centre yes/no (n = centres)	Recruitment	All T2DM (*n*=)	Insulin-dependent (%)			Eating Disorder	Criteria	Questionnaire	Diagnosis		Interview	Diagnosis	
											*n*=	%		*n*=	%
					Total	ED	No ED								
Allison [32]	USA	Yes (*n* = 16)	Open screening and referrals from un-specified healthcare professionals	845	–	–	–	BED	DSM-IV	EDE-Q	47	5.6	EDE^a^	12	1.4
					–	–	–	NES	Clinical assessment^42^	NEQ	71	8.4	NESHI^b^	32	3.8
Çelik [33]	Turkey	No	Diabetes outpatient clinics	152	–	–	–	BED	DSM-IV	–	–	–	SCID-I	8	5.3
Chao [34]	USA	Yes (n = 16)	Open screening and referrals from un-specified healthcare professionals	4572	–	–	–	BED	DSM-5	QEWP-R	54	1.2	–	–	–
Crow [35]	USA	No	Diabetes outpatient clinics	43	–	–	–	BED	DSM-IV	–	–	–	SCID-I	11	25.6
Herbozo [36]	Chile	Yes (−)	Referrals from primary care HCPs	387	0	0	0	BED	DSM-5	QEWP-R	31	8.0	–	–	–
Herpertz [37] 1998	Germany	Yes (*n* = 12)	Inpatients and outpatients	662	51	33	52	BED	DSM-IV	–	–	–	SIAB	18	2.7
Herpertz [38] 2000	Germany	Yes (−)	Diabetes outpatient clinics	322	46	–	–	BED	DSM-IV	–	–	–	SIAB-EX	11	3.4
Hood [39]	USA	No	Endocrinology outpatient clinics	194	–	–	–	NES	DSM-5	NEQ	13	6.7	–	–	–
Kenardy [40]	Australia	No	Diabetes outpatient clinics	50	0	0	0	BED	DSM-IV	QEWP	3	6.0	–	–	–
Mannucci [41]	Italy	No	Diabetes outpatient clinics	156	30	–	–	BED	DSM-IV	–	–	–	EDE	2	1.3

^a^ > 8 objective binge eating episodes in 4 weeks were contacted for an EDE

^b^ NEQ score > 25 were contacted for a NESHI

### Binge eating disorder (BED) and T2DM

Point prevalence data of BED diagnosis was obtained from 6527 participants across 10 studies [32–38, 40, 41]. All studies were cross sectional in design and 5 of these [32, 34, 36–38] were multi-centre studies. One study [32] used two methods of diagnosis and reported point prevalence separately for each. Positive diagnosis according to the EDE-Q questionnaire was then corroborated with the EDE interview. All other studies used exclusively either questionnaires (QEWP-R *n* = 2, QEWP *n* = 1) or interviews (SCID-1 n = 2, EDE n = 1, SIAB n = 1, SIAB-EX n = 1) to establish BED diagnosis. Most studies established diagnosis of BED according to DSM-IV criteria (*n* = 7), while two studies used DSM-5.

Apart from one study [27], which excluded participants taking psychotropic medications, no other studies reported whether participants were taking medications known to influence appetite; such as psychotropic, steroid or anti-diabetes medications like Metformin, DPP-4 inhibitors or GLP-1 agonists. Five studies discussed use of insulin with two studies excluding participants on insulin [36, 40] and in the remaining three studies 30–51% of study participants were on insulin [37, 38, 41]. One study [37] specifically reported insulin use among those with BED (33%) vs. without BED (52%).

### Night eating syndrome (NES) and T2DM

Point prevalence data was obtained from 1039 participants from two studies [32, 39] for NES diagnosis. One study [32] was a multi-centre study. Both studies were cross-sectional, undertaken in the USA and both used NEQ questionnaires to establish NES diagnosis. One used DSM-5 [39] criteria; while the other used published clinical characterisation criteria [42], for diagnosis. One of these studies [32] then used interview to confirm diagnosis, using NESHI, and reported prevalence data according to the alternative methods of diagnosis. Neither of these studies [32, 39] reported insulin use or use of medications that are known to influence appetite.

### Prevalence

Prevalence data is shown in Table 3. The overall point prevalence of BED was 1.2–25.6%. The point prevalence for NES was 3.8% to 8.4% [42]. Using interviews to corroborate questionnaires in one study [32], led to a lesser prevalence of diagnosis of both BED (reduced from 5.6 to 1.4%) and NES (reduced from 8.4 to 3.8%).

### Differences between patients with T2DM with and without BED and NES

#### Body mass index (BMI)

Two studies [33, 36] reported BMI according to absence or presence of BED diagnosis. Both studies showed that BMI was higher in patients with vs. without BED but the difference in the smaller study did not reach statistical significance as shown in Table 4.

**Table 4:** Mean BMI (kg/m2) of Participants With and without BED

Author	With BED			Without BED			Mean Difference (kg/m^2^) [95% CI]	Significance (*p*)
	n	Mean BMI (kg/m^2^)	SD (kg/m^2^)	n	Mean BMI (kg/m2)	SD (kg/m2)		
Çelik [18]	8	33.8	6.8	144	30.8	4.9	3.00 [−1.78, 7.78]	*P* = 0.22
Herbozo [21]	31	34.8	7.9	356	31.4	5.5	3.40 [0.56, 6.24]	*P* = 0.02

The association between NES diagnosis and BMI was reported in one study (*n* = 194, 13 patients with NES) [39]. There was no significant difference between the mean BMI of those with (34.0 kg/m^2^ [SD 8.3]) and without (BMI 35.7 kg/m^2^ [SD 8.3]) a diagnosis of NES (p = ns).

#### Glycaemic control

The same two studies [33, 36] reporting BMI, compared HbA1c between patients with T2DM with and without BED; summarised in Table 5. The larger study [36] showed no difference in HbA1c between patients with and without BED, and the smaller study [33] showed higher HbA1c in patients with BED vs. no BED but this was not statistically significant. It must be noted that in the larger study that showed no difference, the HbA1c was well controlled (7.3%) in both patients with and without BED.

**Table 5:** Mean HbA1c (%) of Participants with and without BED

Author	With BED			Without BED			Mean Difference (%DCCT) [95%CI]	Significance (*p*)
	n	Mean HbA1c (%DCCT)	SD (%DCCT)	n	Mean HbA1c (%DCCT)	SD (%DCCT)		
Çelik [18]	8	8.7	3.3	144	8.1	2	0.60 [−1.71, 2.91]	*P* = 0.61
Herbozo [21]	31	7.3	1.8	356	7.3	2	0.00 [−0.67, 0.67]	*P* = 1.00

Results from Hood et al. [39] found no statistical difference in the HbA1c of those with and without NES, although the number of patients with T2DM was small (8.4% [SD 1.6%] vs. 7.8% [SD 1.6%]; p = ns.

### Ethnicity

No data was available on association between ethnicity and a diagnosis of either BED or NES.

## Discussion

This systematic review showed that BED and NES were common in patients with T2DM, but there was big variation in the prevalence between the studies, which is consistent with the findings of a previous review [11]. This large variation between studies may reflect the different populations studied (primary care vs hospital clinic) as well as the differences in the method used to diagnose BED. Incorporating interviews to the diagnostic process resulted in a lower prevalence of BED and NES compared to questionnaires alone. This difference in the prevalence of BED based on questionnaires vs. interview was also noted previously in patients with morbid obesity who were considered for bariatric surgery [43], which found a lower prevalence of BED using Structured Clinical Interview (SCID) vs. the Questionnaire of Eating and Weight Patterns-Revised (QEWP-R).

The two studies that reported HbA1c in this systematic review showed no statistically significant relationship between BED and HbA1c. One study had very small numbers included, and the larger study had both groups with a mean HbA1c of 7.3%, for patients based in a geographical region in Chile. Findings from this study may not be generalisable to other T2DM populations. And regardless of HbA1c, BED may affect glycaemic variability due to the consumption of large amounts of calories over a short period of time and other behaviours such as prolonged fasting. Hence, the relationship between BED and glycaemic variability needs to be examined, particularly given that there is growing evidence that increased glycaemic variability is associated with increased vascular disease in patients with T2DM [43], independent of HbA1c.

This review found higher BMI in patients with BED, which is consistent with the data from the World Health Organisation showing a high prevalence of obesity amongst patients with BED [9]. However, due to the design of the studies included in our systematic review, it is difficult to ascertain the direction of the relationship between BED and obesity in patients with T2DM, particularly as a bidirectional relationship is biologically plausible. Hence, further longitudinal studies and RCTs are needed to examine the relationships between BED and obesity in patients with T2DM.

### Study limitations

More than half of the included studies were conducted at a single site, reducing the generalisability of the sample. Overall our systematic review showed that there is very limited data available to assess the relationships between BED/NES and diabetes-related outcomes in patients with T2DM. The two studies that measured diabetes-related outcomes and the number of patients with BED and NES included in the analysis were small. Furthermore, all these studies were cross-sectional. Hence causality and direction of relationships could not be ascertained. There is also a lack of data regarding ethnicity, which might play an important role in the relationship between BED/NES and T2DM. Hence, future studies need to take the above-mentioned limitations into account. Moreover, the use of medications known to affect appetite and thus influence eating behaviour was seldom reported.

## Conclusions

Our findings demonstrate that a considerable proportion of adults with pre-existing T2DM have clinical BED and NES. It is therefore important for healthcare professionals working in the field of T2DM to be vigilant regarding the possible diagnosis of BED or NES in their patients. Future studies exploring the impact of BED/NES in the management of T2DM and development of long term diabetes complications are required. The effect of newer anti-diabetes therapies that reduce appetite, and the increasing use of very low calorie diets and bariatric surgery in the management of T2DM, make screening and early diagnosis of these eating disorders even more important.
